# Letrozole as co-treatment agent in ovarian stimulation antagonist protocol in poor responders: A double-blind randomized clinical trial

**DOI:** 10.18502/ijrm.v17i9.5101

**Published:** 2019-09-22

**Authors:** Ashraf Moini, Zohreh Lavasani, Ladan Kashani, Maryam Farid Mojtahedi, Nazila Yamini

**Affiliations:** ^1^Department of Gynecology and ObstetricsArashWomen's HospitalTehran University of Medical Sciences Tehran Iran.; ^2^Breast Disease Research Center (BDRC)Tehran University of Medical Sciences Tehran Iran.; ^3^Department of Endocrinology and Female InfertilityReproductive Biomedicine Research CenterRoyan Institute for Reproductive BiomedicineACECR Tehran Iran.; ^4^Infertility Ward Arash Women's Hospital Tehran University of Medical Sciences Tehran Iran.; ^5^Department of Obstetrics and Gynaecology Islamic Azad University Tehran Medical sciences Branch Tehran Iran.; ^6^IVF Department Embryology Lab Arash Women's Hospital Tehran University of Medical Sciences Tehran Iran.

**Keywords:** Letrozole, Ovarian reserve, Primary ovarian insufficiency, Ovulation induction, Fertilization in vitro, Aromatase inhibitors

## Abstract

**Background:**

Ovarian stimulation (OS) for poor ovarian response (POR) patients is still a major challenge in assisted reproductive techniques. Aromatase inhibitors as co-treatment in antagonist protocol are suggested to these patients, but there are controversial reports.

**Objective:**

To evaluate the effectiveness Letrozole (LZ) as adjuvant treatment in gonadotropin-releasing hormone (GnRH)-antagonist protocol in POR patients undergoing in vitro fertilization/ intracytoplasmic sperm injection cycles.

**Materials and Methods:**

This double-blind randomized clinical trial was conducted in Arash women's hospital. One hundred sixty infertile women with POR based on Bologna criteria were allocated into two groups randomly: LZ + GnRH-antagonist (LA) and placebo + GnRH-antagonist (PA) groups. In the experimental group, the patients received 5 mg LZ on the first five days of OS with 150 IU of recombinant human follicle-stimulating hormone (rFSH) and 150 IUof human menopausal gonadotropin (HMG). The cycle outcomes were compared between groups.

**Results:**

The total number of retrieved oocytes and the metaphase II oocytes in LA-treated group were significantly higher than those in the control group (p = 0.008, p = 0.002). The dosage of hMG used and the duration of OS and antagonist administration in LZ-treated group were significantly lower than those of the control group. The number of patients with no oocyte, in the control group, was higher than the LZ-treated group, and the clinical pregnancy rate in LA-treated group (25%) was higher than the control group (18%); however, the differences were not significant statistically.

**Conclusion:**

Adding 5 mg of LZ to rFSH/hMG antagonist protocol may improve the in vitro fertilization/intracytoplasmic sperm injectioncycle outcome in POR patients.

## 1 Introduction

***Registration ID in IRCT: IRCT201701291952N8***

Poor ovarian response (POR) is a challenging issue for clinicians, since it was associated with high cycle cancellation and low pregnancy rates (1). The ideal controlled ovarian stimulation (COS) options for patients with POR problem remain subjective and not evidence-based (2), and there is still no consensus on the optimal COS protocol in these patients(3). Accordingly, different hormonal manipulations were examined to augment follicular recruitment and to coordinate subsequent antral follicle growth during COH in poor responders (4). “A type of hormonal manipulation included the usage of Letrozole (LZ) as selective, non-steroidal aromatase inhibitors (AIs) blocks androgen conversion to estrogen (5, 6). The LZ-mediated increase ovarian response to stimulation protocol via enhancing in FSH receptors affinity and antral follicles growth by reduction in serum estrogen levels and temporary rise in intraovarian androgen concentrations which cause prolongation of the follicular phase (7, 8). In addition, the decreased serum E_2_ concentration associated with LZ may modify the negative impact of cumulative E_2_ levels on oocyte quality and endometrial receptivity in ART cycles(9)”(10). As an advantage, LZ has no impact on endometrial thickness and complete endometrial recovery before implantation and early embryogenesis; since it has short half-life (approximately 48 hours) and be completely and rapidly cleaned from systemic circulation(11).

“Recently, LZ had been recommended in anovulatory women with high success, and it is popularly applied in the COS (12). The first studies regarding adding LZ in COS protocol for POR patients have reported decreased gonadotropin consumption(7) and and increased number of oocytes retrieved (8)with this method. After that some studies compared LZ-adding antagonist protocol and micro-dose GnRH agonist flare-up protocol in poor responders and have reported controversial results (4). Previous studies regarding this field were different in methodology including the dosage, time of LZ addition, and the initiation time and dosage of gonadotropins (13). Yang and colleagues in a recent study compared the impacts of COH and IVF outcomes of the following three GnRH antagonist protocols: (i) use LZ (5 mg) for five days sequentially overlapping with gonadotropin, (ii) applying LZ (7.5 mg) for three days sequentially with gonadotropin, and (iii) the standard high-dose gonadotropin in a GnRH antagonist protocol in poor ovarian responders (4). They concluded that adding LZ with antagonist protocol is an affordable and preferable protocol”(4). In a recent meta-analysis study, Song and co-workers concluded that the clinical pregnancy rate may be lower with the antagonist/LZ protocol than micro-dose GnRH agonist flare-up protocol for treating poor responders undergoing IVF/ICSI, but large-scale randomized controlled trials are required to evaluate the antagonist/LZ protocol (14).

Consequently, this study was designed as a double-blind clinical trial to assess the potential effect of LZ as an adjuvant drug to improve the outcomes of standard GnRH antagonist stimulation protocol in patients with POR diagnosis.

## 2 Materials and Methods

### 2.1 Patient selection

In this randomized clinical trial which conducted at the infertility center of Arash Women’s Hospital, all women with POR diagnosis who underwent in vitro fertilization/intracytoplasmic sperm injection and fresh embryo transfer (IVF/ICSI-ET) cycles were assessed from 3rd February 2017 to 7th September 2017. POR was defined according to the Bologna criteria and the patients who had at least two of the following three criteria were included: (i) a prior history of POR (retrieved oocytes ≤ 3) treatment cycle by the conventional COS, (ii) advanced maternal age (≥40 yr) or any other anamnestic risk factors POR (e.g., a history of ovarian surgery, previous chemotherapy, genetic abnormalities, shortening of the menstrual cycle), and (iii) abnormal ovarian reserve test (i.e., antral follicle count (AFC) < 5–7 follicles or anti-Mȕllerian hormone (AMH) < 0.5–1.1 ng/ml). Exclusion criteria were as follows: premature ovarian failure diagnosis, donor/recipient treatments, metabolic or endocrine disorders including hyperprolactinemia and hypo/hyperthyroidism, endometriosis, body mass index (BMI) > 30 kg/m^2^, and azoospermic male partner.

The previous COS was considered by a minimum of two or more months to prevent any potential source of error. Block randomization method with blocks number six was conducted by statistics advisor using STATA software version 13 for randomization of the patients into two groups. Only the statistics advisor was informed regarding the random allocation list of patients. In order to hide the random allocation process, a total of 160 envelopes of a single drug form were prepared, a random 10-digit-numbered code was decided, and a framework was written that was the relevant drug identification number, with only the methodologist being aware of the design of the code. As soon as the patient's eligibility was determined by clinical specialists, the statistics advisorprovided the envelope with them and the grouping type was selected on the basis of what was inserted in the envelope.All placebo tablets were produced by Iran hormone company (Tehran, Iran), which was approved by the Food and Drug Administration of Iran. The appearance of the placebo (containing 1 mg folic acid, Iran hormone, Tehran, Iran) was indistinguishable in color, shape, size, and smell from LZ tablets. On the basis of Ebrahimi *et al*.’s study (14), since folic acid (1 mg) tablet is administrated preconceptionally to prevent the fetal nervous system abnormalities in all infertile patients and it has no effect on the OS outcome, therefore using additional folic acid (1 mg) in the control group as placebo is ethical and logical option. The person evaluating the outcomes was the third person who was unaware of the random allocation process and the type of the treatment. Data analysis was carried out by a statistician who was unaware of all the processes of study.

### 2.2 Treatment protocols

The ovarian stimulation for all study participants was a flexible regimen of GnRH-antagonist protocol. The ovarian quiescence was defined by detecting the serum estradiol (E_2_) concentrations < 60 pg/mL and absence of ovarian cysts > 10 mm diameter on vaginal ultrasound scans on day 2 of menstrual cycle. The baseline serum follicle-stimulating hormone (FSH), luteinizing hormone (LH), E_2_, and progesterone (P) levels were measured on day 2 or 3 of menstrual cycle before starting gonadotropin stimulation. The eligible patients on 2nd or 3rd day of menstrual cycle were randomly allocated into two groups in a 1:1 ratio by either adding LZ or placebo to GnRH-antagonist stimulation protocol. In the experimental (LZ)group, the patients received 5 mg LZ (Letrofem®; Iran hormone, Tehran, Iran) on the first five days of OS with 150 IU of recombinant human FSH (Cinnal-f, Cinagen) and 150 IUof human menopausal gonadotropin (HMG) (Menogan, Ferring). In the control group (placebo), the patients received 150 IU of rFSH and 150 IU of HMG with placebo (containing 1 mg folic acid, Iran hormone, Tehran, Iran). The follicular growth was evaluated by the serial vaginal ultrasound (sonographic device: Phillips, affinity 70) and the measurements of serum E_2_ level. The dosage of rhFSH was modified individually on the basis of the ovarian response. The GnRH antagonist, cetrorelix (Cetrotide ®, Serono International, Geneva, Switzerland), 0.25 *μ*g/day was subcutaneously administrated when follicle(s) ≥ 13 mm diameter was observed and continued until the day of triggering of ovulation. The serum P and E_2_ levels were measured at the day of human chorionic gonadotropin (hCG) injection. The final stage of oocyte maturation was triggered by 10,000 units of hCG (Choriomon, IBSA, InstitutBiochimique SA),when at least two follicles with  ≥ 18 mm in diameter was observed and the serum E_2_ concentration ≥ 500 pg/mL was measured. The cycle was cancelled on poor response, if these criteria were not achieved after 10–12 days of stimulation. Transvaginal ultrasound-guided oocyte retrieval was carried out 34–36 hrs. after the oocyte triggering. The fertilization was done by conventional ICSI. The obtained embryos at cleavage stage were replaced under an ultrasound scan guidance by an embryo transfer catheter (Guardia™, Access ET Catheter, Cook Medical), two or three days after the oocytes retrieval. Embryo quality was defined based on the number and regularity of blastomeres and the degree of embryonic fragmentation. All patients received luteal phase support in the form of 400 mg vaginal progesterone suppository twice daily (Cyclogest, Actavis, Barnstaple, UK) starting on the oocyte retrieval evening, and it was continued for 10 weeks in patients with a positive pregnancy test. A serum ß-hCG analysis was performed 14 days after the ET, and the clinical pregnancy was detected by observation of gestational sac with a heartbeat using ultrasound scan 7–10 days later.

The number of oocytes retrieved and the number of oocytes MII were considered as primary outcomes in the present study. The secondary outcomes were fertilization, implantation, cycle cancelation, and clinical pregnancy rates, total gonadotropin dose, duration of OS, the endometrial thickness and peak serum E2 levels on trigger day, and a total number of obtained embryos.

### 2.3 Ethical consideration

The trial protocol was approved by the Institutional Review Board and the Ethics Committee of the Tehran University of Medical Sciences, Tehran, Iran (Ethics code: IR.TUMS.MEDICINE.REC.1395.1245). The written informed consent was obtained from the eligible patients before entering the study.

### 2.4 Statistical analysis

It was estimated 103 subjects were required in each study group based on Ebrahimi and colleagues study (10) and using NCSS-PASS software (version 2007; NCSS Inc., Kaysville, UT, USA) with α = 0.05 and 80% power, so a pilot trial was designed by 80 women in each group according to the timetable. After the study was performed, the post-hoc power was calculated as 70%. The statistical analysis was performed by Statistical Package for the Social Sciences (SPSS) (version 20, SPSS Inc., Chicago, Illinois, USA). The independent *t*-test and Chi-square test were used for comparison of qualitative and quantitative variables between groups respectively. Descriptive data are reported as mean ± standard deviation (SD) or number (percent) as appropriate. Statistical significance level was considered at p < 0.05.

## 3 Results

**Figure 1:**
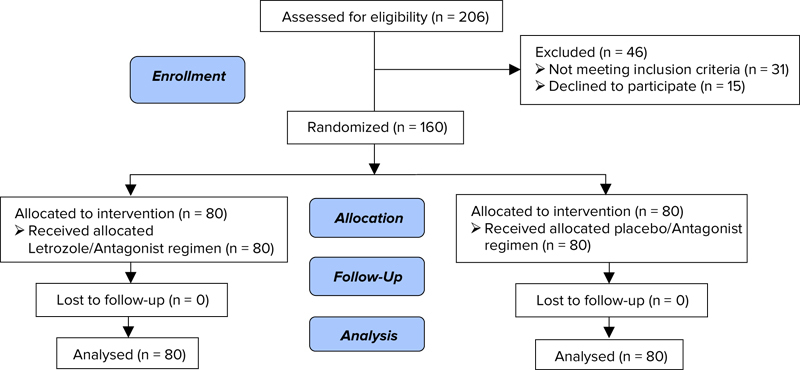
Recruitment follow-up and dropouts during the study (Consort flowchart).

Finally, 206 patients with POR diagnosis were evaluated and 46 women were excluded due to the exclusion criteria (n = 35) and not satisfied to participate (n = 11). No case of dropout was occurred in both groups; therefore, the data of 160 participants were analyzed (Figure 1). Table I compares the demographic and clinical characteristics of study participants between groups. There were no significant differences between groups in terms of female age, duration of infertility, BMI, serum AMH, basal FSH, and LH levels, and AFC before starting the COS protocols. Sixty women in LZ group (75%) and sixty-four women in the control (placebo) group (80%) had at least one history of POR with conventional long-GnRH agonist protocol.

The outcomes of treatment cycles are reported in Table II. The dosage of hMG used and the OS duration and antagonist administration in LZ-treated group were lower than those of in the placebo group significantly. There were no differences in the means of the serum P level on hCG administration day and the endometrial thickness between groups. The mean concentration of serum E2 at trigger day was found to be lower in LZ co-treated group than that of the control group; however, it was not statistically significant (p = 0.08). The mean number of oocytes retrieved, the metaphase II oocytes in LZ co-treated group were significantly higher than those of the control group. The fertilization rate, the mean number of obtained embryos, top quality and transferred embryos were comparable between groups. In the control group, the number of patients with no oocyte result was higher; however, the difference between the two groups did not reach the statistical significance level (p = 0.1). There were no significant differences in implantation and clinical pregnancy rates between the groups (p = 0.1 and p = 0.3, respectively).

## 4 Discussion

The results of the current trial indicates the number of oocytes retrieved and that MII oocytes were improved and a lower dosage of hMG and antagonist administration and a shorter duration of stimulation were required with adding LZ. In addition, the number of cases with no oocyte result in the control group was twice. The trend toward improvement in implantation and clinical pregnancy rates were observed in the LZ-treated group; however, it was not statistically significant.

At first, Garcia-Velasco and co-workers assessed the effect of AIs on ovarian response and IVF outcomes in patients with POR, using an OCP/GnRH-antagonist protocol and reported that LZ-treated patients had significantly higher levels of follicular fluid testosterone, androstenedione, and more oocytes retrieved and a higher IR, despite similar doses of gonadotropins (8). To the best of the knowledge, there were nine clinical trials that evaluated the effect of adding LZ to antagonist protocol (10, 15–22); of these, four trials compared it with micro-dose GnRH agonist flare-up protocol (16, 18, 19, 22), three studies with antagonist protocol without placebo (15, 17, 21), one trial with placebo/antagonist protocol (10), and one study with clomiphene/antagonist protocol as control group (20).

In agreement to the present results, Ozmen and colleagues evaluated the effect of adding LZ (5 mg/day) to a fixed dosage (450 IU/day) of r-hFSH on intra-ovarian androgens and cycle outcomes but controls with the same r-hFSH dosage alone (15). It was concluded that adjunctive LZ administration is beneficial since it reduces both cycle cancellation rate and cost without an adverse effect on the outcome (15). Similarly, Bastu and co-workers compared three different gonadotropin doses with or without the addition of LZ during the antagonist protocol in patients with POR. It was found that a mild stimulation by using LZ was effective as well as stimulation with higher doses of gonadotropins alone in these patients (21). Elsewhere, Mohsen and El Din concluded that adding LZ in antagonist protocol and micro-dose GnRH agonist flare-up protocol had same clinical outcomes; however, the former was more affordable and patient-friendly in POR (18). However, other studies did not find any positive effect from adding LZ to conventional GnRH antagonist protocol and reported some conflicting results (10, 16, 17, 19, 22). We postulated that the controversial results between the studies might be due to the different methodology and using different criteria and cut-off values for ovarian reserve tests to define the POR. Moreover, it may be attributed to the different sample sizes, different doses of LZ used (2.5 versus 5 mg) and the different starting days (23). In a double-blind clinical trial with same inclusion criteria, Ebrahimi and colleagues found no improvement in clinical outcomes in POR patients; although the sample size of this study was low, it can affect the power of the conclusion (10).

### 4.1 Strengths and limitations of the study

It should be mentioned that the present study had some limitations and some strong points. This study has low power due to difficulties of the participants enrolment and a low number of the eligible patients same as previous studies in this field (5, 7, 8, 10, 15, 24, 25). Although, the main outcome of the present study were in parallel to the previous large retrospective and prospective non-randomized studies (5, 15, 24, 25) in terms of gonadotropins consumption dosage, E2 level, and pregnancy rate in the LZ co-treated group, the present study is one of the clinical trials with large sample size and proper patient selection based on “Bologna criteria.” In the present study, patients are POR and have already had an IVF failure, PGS needs to be performed for reducing fertility failure as a result of fetal abnormalities, but these patients were not willing to apply this approach to their embryos due to cultural and religious issues.

## 5 Conclusion

In conclusion, according to the results of the current clinical trial, adding LZ to the rFSH/hMG antagonist protocol may improve the outcome of the IVF/ICSI cycles in patients with POR. In addition, considering the evidence provided with previous studies and current trial, we suggested designing future studies to evaluate the effect of adjunctive use of low-dose LZ in conventional OS regimes on IVF/ICSI outcomes in patients with unexplained infertility, endometriosis, and PCOS diagnosis.

**Table I:** The comparison ofdemographic and clinical characteristics of study participants between groups

Variables^*^	Letrozole group	Control group	p-value
Female age (yr)*	37.2 ± 3.3	36.5 ± 3.7	0.2
Female age groups^**^			
≤ 35 yr old	26 (32.5)	30 (37.5)	0.6
> 35 yr old	54 (67.5)	50(62.5)	
Body mass index (kg/m^2^)^*^	25.9 ± 3.7	26.3 ± 3.4	0.5
Duration of Infertility (yr)^*^	7.3 ± 6.1	6.7 ± 5.1	0.7
No. of couple with primary infertility^**^	69 (86.2)	65 (81.2)	0.8
Early follicular phase FSH (IU/L)^*^	8.7 ± 3.6	8.0 ± 2.9	0.1
Early follicular phase LH (IU/L)^*^	6.0 ± 3.5	6.4 ± 5.9	0.6
Early follicular phase E_2_ (pg/mL)^*^	58.9 ± 6.9	57.3 ± 6.1	0.1
TSH (IU/mL)^*^	2.4 ± 1.4	2.3 ± 1.0	0.6
Prolactin(ng/mL)^*^	17.4 ± 11.0	18.7 ± 8.7	0.4
AMH (ng/mL)^*^	0.65 ± 0.35	0.73 ± 0.31	0.1
Antral follicle count^*^	5.1 ± 2.0	4.8 ± 1.5	0.2
No. of previous failed cycles^*^	1.0 ± 0.9	1.1 ± 0.6	0.5

* Variables were compared between groups and presented as mean ± SD;

** data were compared between groups and presented as n (%)

p ≤ 0.05 was considered statistically significant

No.: Number; FSH: Follicle-stimulating hormone; LH: Luteinizing hormone; TSH: Thyroid-stimulating hormone; AMH: Anti-müllerian hormone; E_2_: estradiol; P: Progesterone

**Table II:** . The comparison of ovarian stimulation and cycle outcomes between the study groups

Variables^*^	Letrozole group	Control group	p-value
Total rFSH dose (IU)^*^	1433.4 ± 324.4	1490.6 ± 273.0	0.2
Total hMG dose (IU)^*^	1386.5 ± 237.7	1482.1 ± 256.2	0.01
Total gonadotropins dose (IU)^*^	2820 ± 522.4	2972.8 ± 512.1	0.06
Duration of stimulation (Day)*	8.9 ± 1.4	9.7 ± 1.3	0.001
Peak E_2_ level at trigger (pg/mL) *	776.3 ± 74.9	796.3 ± 72.2	0.08
Serum progesterone at trigger (ng/mL) *	0.87 ± 0.25	0.93 ± 0.26	0.1
Duration of antagonist administration (Day)*	3.5 ± 1.1	4.4 ± 1.4	< 0.001
No. of retrieved oocytes*	3.6 ± 2.5	2.7 ± 1.4	0.008
No. of cases with no oocyte result**	4 (5)	9 (11.1)	0.1
No. of metaphase II oocytes *	3.0 ± 2.2	2.0 ± 1.3	0.002
Fertilization rate (%)*	79.1 ± 32.5	74.1 ± 33.6	0.3
No. of obtained embryos*	1.9 ± 1.4	1.7 ± 1.1	0.4
Top quality embryo n (%)*	1.8 ± 1.1	1.5 ± 1.0	0.1
No. of cases with no embryo result **	12 (15)	11 (13.7)	0.9
No. of embryos transferred*	1.9 ± 1.0	1.8 ± 0.8	0.6
Endometrial thickness at the trigger(mm)*	8.6 ± 1.8	8.8 ± 1.3	0.3
Implantation rate *	15.8 ± 32.2	8.3 ± 19.5	0.1
Clinical pregnancy/ET (%)**	16/64 (25)	11/60 (18.3)	0.3

* Variables were compared between groups and presented as mean ± SD;

** data were compared between groups and presented as n (%)

p ≤ 0.05 was considered statistically significant

hMG: Human menopause gonadotropin;

rFSH: Recombinant follicle-stimulating hormone; No.: Number

## Conflict of Interest

There are no conflicts of interest to declare.
